# Menstruating from the umbilicus as a rare case of primary umbilical endometriosis: a case report

**DOI:** 10.1186/1752-1947-3-9326

**Published:** 2009-12-10

**Authors:** Pallavi V Bagade, Mamdouh M Guirguis

**Affiliations:** 1 Department of Obstetrics and Gynaecology, Wansbeck General Hospital, Woodhorn Lane, Ashington NE63 9JJ, Northumberland, UK; 2 Department of Obstetrics and Gynaecology, North Tyneside General Hospital, Rake Lane, North Sheilds NE29 8NH, Tyne and Wear, UK

## Abstract

**Introduction:**

Endometriosis is a common gynecological condition and presents mainly with involvement of the pelvic organs. Extrapelvic presentations in almost all parts of the body have been reported in the literature. However, umbilical endometriosis that is spontaneous or secondary to surgery is uncommon and accounts for only 0.5% to 1% of all endometriosis cases.

**Case presentation:**

A 35-year-old Caucasian woman presented with umbilical bleeding during periods of menstruation. Her umbilicus had a small nodule with bloody discharge. An ultrasound was performed and a diagnosis of possible umbilical endometriosis was thus made. The nodule shrunk in response to gonadotropin-releasing hormone analogues but continued to persist. The patient underwent a wide local excision of the nodule with a corresponding umbilical reconstruction. Histopathology confirmed the diagnosis of umbilical endometriosis. The patient was asymptomatic at follow-up, but nevertheless warned of the risk of recurrence.

**Conclusions:**

Pelvic endometriosis is a common condition, but the diagnosis of primary umbilical endometriosis is difficult and differentials should be considered. This case strongly suggests that a differential diagnosis of endometriosis should be considered when an umbilical swelling presents in a woman of reproductive age.

## Introduction

Endometriosis, a term first used by Sampson, is the presence of endometrial glands and stroma outside the uterine cavity and musculature [1]. It affects 7% to 10% of women in the reproductive age group [2]. It commonly occurs in the pelvic organs, especially the ovaries, the uterosacral ligaments and the pouch of Douglas. Women with endometriosis often present with dysmenorrhea, menorrhagia, pelvic pain and infertility.

Extragenital endometriosis is less common, but has been described in almost every area of the female body including the bowel, bladder, lungs, brain, umbilicus, and surgical scars [3]. Due to its varied presentations, endometriosis remains a difficult condition to diagnose and treat.

Umbilical endometriosis represents 0.5% to 1% of all cases of extragenital endometriosis. It usually occurs secondary to surgical scars, but very rarely presents as primary umbilical endometriosis [4,5]. We report one such rare case of spontaneous, primary umbilical endometriosis.

## Case presentation

A 35-year-old Caucasian parous woman presented to the clinic with symptoms of spontaneous and periodic bleeding from the umbilicus for four months. The bleeding would start two days before her menses and continue for the entire duration of her period. It was accompanied by pain and swelling in the umbilical area.

The patient had regular, heavy and painless menstrual periods and did not wish for any treatment for such. She had two previous spontaneous vaginal deliveries and had no history of abdominal pain, dyspareunia or infertility. She was not using any form of hormonal contraception. Her medical history was not significant and she never had any abdominal surgeries.

Clinical examination revealed that the patient had a 2 cm × 2 cm firm nodule at the umbilicus, which appeared to be covered by a reddish brown discharge. Suspecting that she had an infection, the patient was swabbed and given a five-day course of oral broad-spectrum antibiotics. She showed up on check up two months later with no relief of symptoms. She then underwent an ultrasound scan that showed a 15-mm thin-walled cyst, approximately 5 mm below the skin surface. The key clinical feature that led to the correct diagnostic hypothesis of umbilical endometriosis was the temporal association of the bleeding with her menstrual period.

The patient was offered both medical and surgical management and she opted to have depot injections of Zoladex (AstraZeneca UK, Goserelin acetate, 3.6 mg subcutaneously, monthly). The swelling continued to persist in spite of three doses of Zoladex, and the patient then requested surgical excision. The risk of recurrence and scar endometriosis were explained to her.

**Figure 1:**
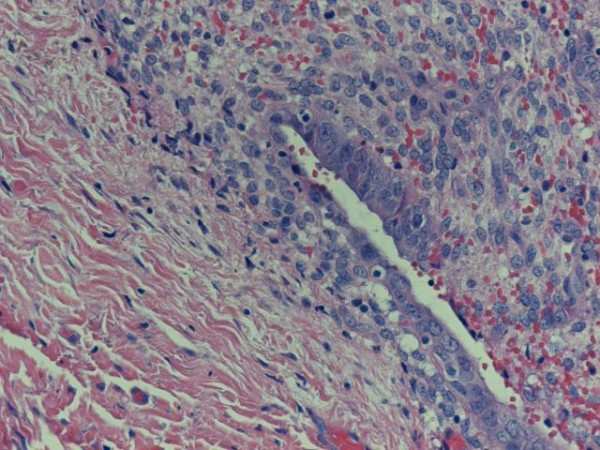
**Umbilical endometriosis: endometriotic glands with metaplasia of the mucinous type and extravasation of the mucinous secretion into the adjacent stroma**.

The patient successfully underwent excision of the nodule with accompanying umbilical reconstruction. Histology confirmed the diagnosis of endometriosis and revealed the presence of endometriotic glands with mucinous type metaplasia and extravasation of the mucinous secretion into the adjacent stroma (Figure 1). No epithelial atypia was seen and the excision appeared complete. The patient was seen six weeks after the surgery and found to be asymptomatic with a normal umbilicus. Before being discharged, the patient was again reminded of the risk of recurrence.

## Discussion

The deposition of fragments of uterine endometrium in the skin is a well recognized, although uncommon, phenomenon (0.5% to 1% of extragenital endometriosis). Umbilical endometriosis was first described in 1886 and since then more than 100 cases have been described [4]. Majority of these cases occurred secondary to surgical, commonly laparoscopy, scars. An umbilical endometriotic lesion without surgical history is a rare condition [4,5]. Some case reports have also described the presence of umbilical endometriosis during pregnancy [6].

There has been great speculation about the pathogenesis of this phenomenon and several theories have been proposed. Latcher has classified these theories into three main categories: the embryonal rest theory, which explains endometriosis adjoining the pelvic viscera by Wollfian or Mullerian remnants [4,5]; the coelomic metaplasia theory, which states that the embryonic coelomic mesothelium dedifferentiates into endometrial tissue under stimulus such as inflammation or trauma [7]; and the migratory pathogenesis theory, which explains the dispersion of endometrial tissue by direct extension, vascular and lymphatic channels, and surgical manipulation. Still others suggest cellular proliferation of endometrial cells from initial extraperitoneal disease along the urachus [8,9]. The real mechanism still remains a mystery.

These patients are usually in the reproductive age group and present commonly with swelling, pain, discharge or cyclical bleeding from the umbilicus. There may be associated symptoms of coexistent pelvic endometriosis. These lesions are usually bluish-black in colour and become painful, larger and bleed about the time of menses. They range in size from 0.5 cm to 3 cm, but can enlarge to even more enormous sizes [4].

While the diagnosis is primarily clinical, magnetic resonance imaging (MRI) can be useful in evaluating patients with suspected endometriosis. Endometriomas appear homogeneously hyperintense on T1-weighted sequences [10]. MRI also has an advantage over laparoscopy for evaluating pelvic and extraperitoneal diseases, as well as lesions concealed by adhesions.

Histological findings are characterized by irregular glandular lumina embedded in the stroma with a high cellular and vascular component resembling the stroma of functional endometrium. A fairly recent study has suggested a distinctive dermatoscopic feature in cutaneous endometriosis -- that of comprising small red globular structures called 'red atolls' [11].

Differential diagnosis of umbilical nodules should include pyogenic granuloma, hernia, residual embryonic tissue, primary or metastatic adenocarcinoma (Sister Joseph's nodule), nodular melanoma, and cutaneous endosalpingosis.

Surgical excision of the lesion with sparing of the umbilicus is the preferred treatment of pelvic endometriosis [7]. In severe cases or in the presence of pelvic endometriosis, hormonal therapy in the form of danazol or GnRH analogues can be given to the patient [12]. In our case the lesion was excised and histology confirmed the diagnosis. Although simultaneous laparoscopy has been recommended for pelvic endometriosis, this was not done because our patient was asymptomatic. Although local recurrence is uncommon, the patient has been warned of the risk of scar endometriosis and of recurrence.

## Conclusions

Endometriosis is a common gynaecological disease; however, primary umbilical endometriosis is very rare. Making a diagnosis is difficult and other causes of umbilical lesions should be considered. Surgical excision is the standard treatment of this condition.

## Abbreviations

MRI: magnetic resonance imaging; GnRH: gonadotropin releasing hormone.

## Consent

Written informed consent was obtained from the patient for publication of this case report and any accompanying images. A copy of the written consent is available for review by the Editor-in-chief of this journal.

## Competing interests

The authors declare that they have no competing interests.

## Authors' contributions

PB was a major contributor in collecting data, writing and preparing the manuscript. MG performed the surgical excision and was involved in editing the manuscript. All authors read and approved the final manuscript.
